# Prolonged detection of complete viral genomes demonstrated by SARS-CoV-2 sequencing of serial respiratory specimens

**DOI:** 10.1371/journal.pone.0255691

**Published:** 2021-08-05

**Authors:** Neta S. Zuckerman, Efrat Bucris, Oran Erster, Michal Mandelboim, Amos Adler, Saar Burstein, Noam Protter, Moran Szwarcwort-Cohen, Ella Mendelson, Orna Mor

**Affiliations:** 1 Central Virology Laboratory, Ministry of Health, Chaim Sheba Medical Center, Ramat-Gan, Israel; 2 Department of Epidemiology and Preventive Medicine, School of Public Health, Sackler Faculty of Medicine, Tel-Aviv University, Tel-Aviv, Israel; 3 Clinical Microbiology, Tel-Aviv Sourasky Medical Center, Tel-Aviv, Israel; 4 IDF Medical Corps, Ramat-Gan, Israel; 5 Virology Laboratory, Rambam Health Care Campus, Haifa, Israel; Universidad Nacional de la Plata, ARGENTINA

## Abstract

Accurate and timely diagnosis of severe acute respiratory syndrome coronavirus 2 (SARS-CoV-2) is clinically essential, and is required also to monitor confirmed cases aiming to prevent further spread. Positive real-time PCR results at late time points following initial diagnosis may be clinically misleading as this methodology cannot account for the infection capabilities and the existence of whole genome sequences. In this study, 47 serial respiratory samples were tested by Allplex-nCoV test (Seegene), a triplex of three assays targeting the SARS-CoV-2 RdRP, E and N genes and subsequently assessed by next generation sequencing (NGS). COVID19 patients were tested at an early stage of the disease, when all these viral gene targets were positive, and at an advanced stage, when only the N gene target was positive in the Allplex-nCoV test. The corresponding NGS results showed the presence of complete viral genome copies at both early and advanced stages of the disease, although the total number of mapped sequences was lower in samples from advanced disease stages. We conclude that reduced viral transmission at this late disease stage may result from the low quantities of complete viral sequences and not solely from transcription favoring the N gene.

## Introduction

Severe acute respiratory syndrome coronavirus 2 (SARS-CoV-2), first identified in China in December 2019 [1], causes corona viral disease (COVID-19) and was declared a pandemic by the world health organization on March 2020 [2]. Accurate and timely diagnosis of SARS-CoV-2 is crucial to identify infected individuals, monitor confirmed cases and prevent further spread.

Real-time reverse transcription polymerase chain reaction (RT-PCR) that detects the presence of specific SARS-CoV-2 RNA molecules (“targets”) in nasopharyngeal swab samples, is considered the gold-standard methodology for diagnosis of this infection. This method measures the accumulation of a specific fluorescent signal in comparison to background signal level. This is represented by the number of amplifications from which the signal is detectable, also referred to as the cycle threshold (Ct) value [3]. RT-PCR results are usually qualitative, yielding a positive or negative answer, however as Ct levels are inversely proportional to the target RNA copies, it can also be used as a quantitate analysis [4]. High viral titers / low Ct values were also found to correlate with culture-positive samples [5].

A widely used RT-PCR-based assay in Europe and other countries (e.g. Brazil, Canada and Korea) is Allplex 2019-nCoV assay (Seegene), which simultaneously detects the RdRP, N and E SARS-CoV-2 gene targets [6, 7]. In Israel, Allplex 2019-nCoV RT-PCR assay was used by the vast majority of diagnostic laboratories during the first COVID-19 wave in February-May 2020, and is still used by most of them today. Using the assay, it is commonly reported that initial phases of the disease are characterized by low Ct values for all three genes and in later time points, the SARS-CoV-2 viral load decreases and the Ct values increase accordingly. However, in a subset of samples taken late after initial diagnosis (even >14 days), the E and RdRP genes are not detected while the N gene target remains exclusively positive, albeit with high Ct values compared to earlier time points. Indeed, the viral N protein, involved in key regulatory functions in addition to encapsulating the viral genome [8], is the most abundantly expressed transcript in SARS-CoV-2 [9]. We therefore hypothesized that only the N gene transcripts remain highly abundant at late time-points and that the Allplex assay is sensitive enough to identify them, while whole genome viruses are degraded, as already suggested by others [10].

Accurate assessment of the viral transmission is crucial for providing evidence-based public health policies, e.g. determining rules for quarantine length, return to work and contact tracing. Positive RT-PCR results do not reflect the rate of viral transmission as it is not a functional assay and thus detection of viral RNA by this approach does not determine infectivity [4, 11]. Culturing of virus from patients recovering from the disease, with high SARS-CoV-2 Ct values or those positive for the N-gene only, is also expected to fail and is also impractical for routine use [5].

To better understand the Allplex assay results, we sequenced SARS-CoV-2 whole genomes and compared genomic coverages between serial respiratory samples obtained from the same patient at different time-points post diagnosis. Specifically, we examined whether the absence of the E and the RdRP genes in the RT-PCR assay in recovering patients represent disintegrated RNA reminiscent of the virus.

## Materials and methods

Swab samples from 52 individuals (150 samples total) were tested using the Allplex 2019-nCoV assay (Seegene Inc., Korea) according to the manufacturer’s instructions. Samples included in this analysis were those collected at diagnosis and at different time-points post initial diagnosis (Tables 1 and S1). To assess the performance of this Allplex assay, four other SARS-CoV-2 positive samples were diluted by a SARS-CoV-2 negative sample and tested using this rt-PCR assay (S2 Table).

**Table 1:** Summary of the samples (n = 150) included in this study.

Laboratory	# Patients	Age	Gender	# Samples	N Ct	RdRP Ct	E Ct
ICVL	4	14–42	M (2), F (2)	16	22–38	22–40 + ND	20–39 + ND
ICH	7	41–86	M (3), F (4)	24	20–39	18–39 + ND	17–33 + ND
IDF	5	19–46	M (4), F (1)	15	25–39	23–37 + ND	22–33 + ND
RAM	36	19–90	M (28), F (8)	95	15–40	15–38 + ND	12–37 + ND

Laboratory of origin, number patients, age range, gender, number of samples (included are samples collected from at least two time-points per patient), and ranges of Ct values obtained using the Allplex-2019 nCov assay for the N, RdRP and E reactions are shown. Target-specific Ct values for each reaction for all samples are detailed in S1 Table. M, Male, F, Female; ICVL: Israel central virology laboratory, ICH: Ichilov hospital microbiology laboratory, IDF: Israel defense force laboratory, RAM: Rambam hospital virology laboratory; ND: not detected.

SARS-CoV-2 next generation sequencing (NGS) analysis was performed on 45 samples of 16 patients selected, based on availability, and on having patient-matched samples from at least two time-points, taken at least 5 days apart (S1 Table). SARS-CoV-2 specific primers designed to capture SARS-CoV-2 whole genome (ARTIC network V3 primer set, https://artic.network/ncov-2019) were used for amplification and preparation of libraries with NexteraXT as per the manufacturer’s instructions, and sequenced on NextSeq (Illumina, CA, USA). Sequencing coverage, i.e. the fraction of positions covered by at least 5 reads, was calculated for full gene segments containing the N gene, E gene and Nsp12 region (containing the RdRP gene) and for the whole viral genome in each sample. All sequences are available in the global science initiative on sharing all influenza data (GISAID), accession numbers are listed in S1 Table.

The study was conducted according to the guidelines of the Declaration of Helsinki, and approved by the Institutional Review Board of the Sheba Medical Center institutional review board (7045-20-SMC). Patient consent was waived because the study used remains of clinical samples and the analysis used anonymous clinical data.

## Results

**Fig 1:**
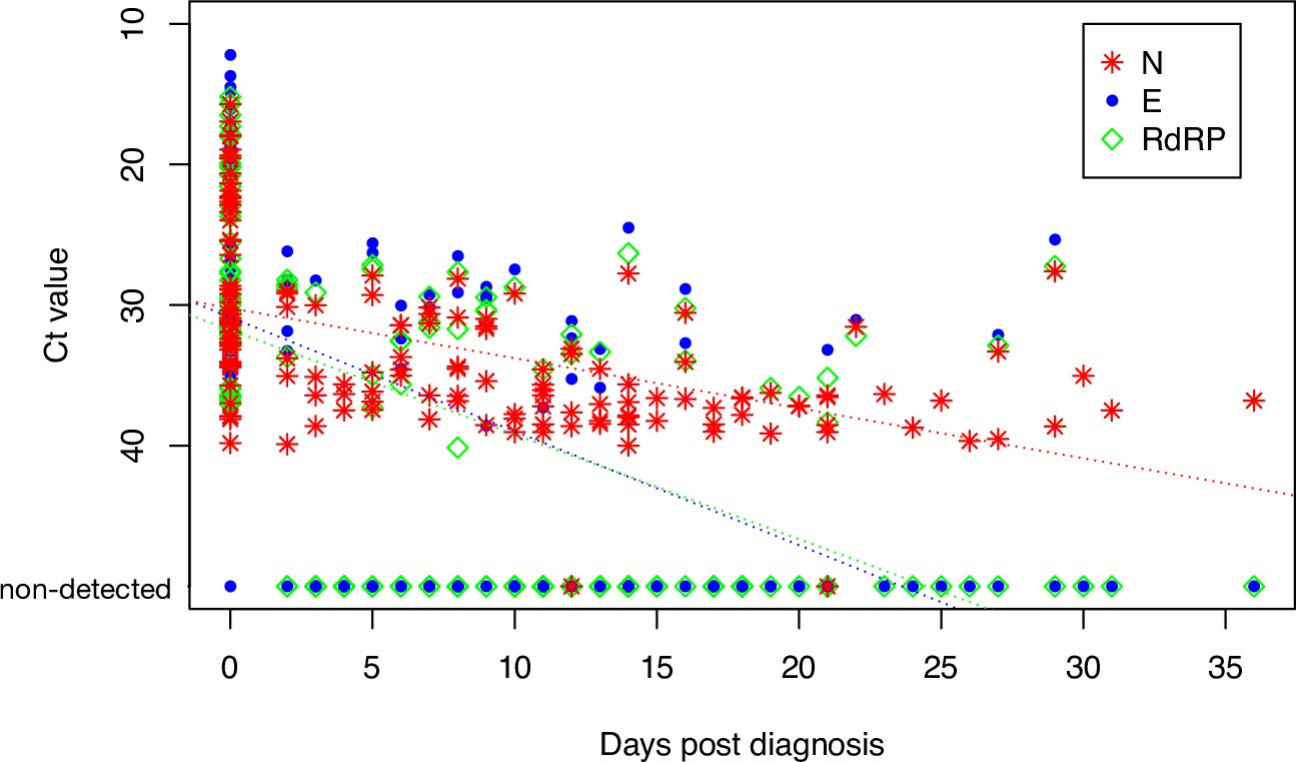
Ct values per days post diagnosis. Ct values of N, E and RdRp reactions, obtained using the Allplex 2019-nCoV assay in patient serial samples. Negative reactions (Ct value > 40) are denoted. Dotted lines represent linear regression per each gene, with the coefficient of determination (r2) and T-test p value (p) denoted in the figure legend. The insert summarizes the days post diagnosis in which each gene is detected (Ct values>40 not included).

First, we assessed the RT-PCR results obtained from 150 samples from 52 individuals. While the Ct values of all targets- RdRp, E and N were low at diagnosis, linear regression of serial patient’s samples demonstrates different rate of change in Ct values for RdRp and E compared to the N gene targets. In all samples, the E and RdRp genes were detected with relatively low Ct values upon initial diagnosis and remained detectable up to day 16 post-diagnosis. The N gene, however, remained detectable in samples taken at late time-points and could be identified in up to day 31 post diagnosis (Fig 1). Similarly, N gene target was the only gene remaining detectable in positive samples following serial dilution (S2 Table).

**Fig 2:**
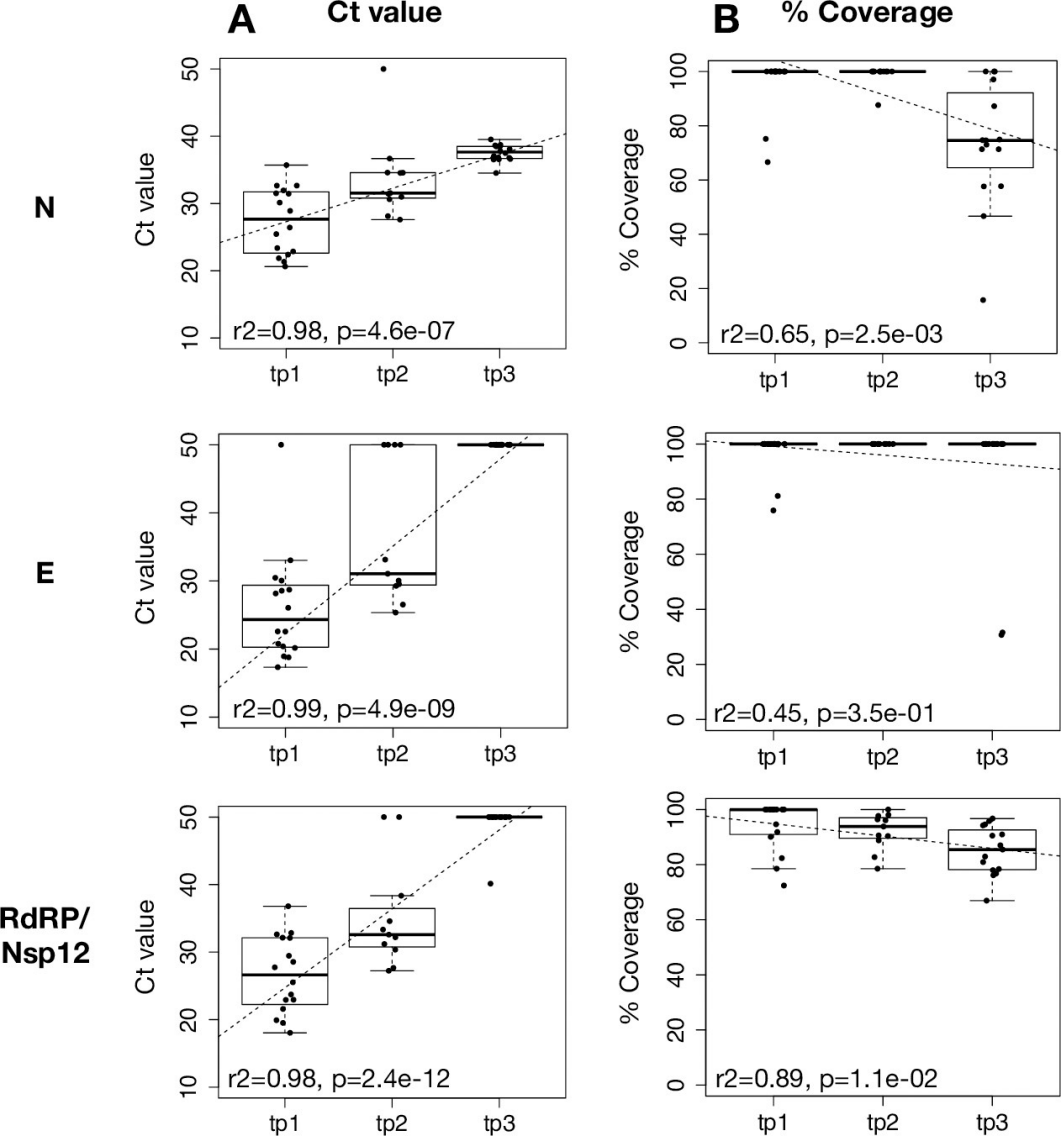
Genome coverage and viral mapped reads. Ct values of N, E and RdRp reactions obtained using the Allplex 2019-nCoV assay, plotted against SARS-CoV-2 complete genome coverage (% coverage, red triangles) and number of reads mapping to the viral genome normalized by the total number of reads per sample (% mapped reads, blue squares). Dotted lines represent linear regression per each measured quantity, with the coefficient of determination (r2) and T-test p value (p) denoted in the figure legend. The inserts summarize the Ct values obtained for each of the time points (tp1-3).

Fig 2 summarizes the NGS results. Both SARS-CoV-2 whole genome coverage, represented by the percent of positions in the genome that have sequencing depth of >5, and reads mapping to the viral genome, (normalized by the total number of reads per sample), significantly decreased as Ct values increased and at the later time-points post diagnosis (Fig 2). Individual SARS-CoV-2 genes, including E, N and RdRp, exhibited various patterns of coverage over time, with some genes remaining fully covered in all time points (including E and Nsp12/RdRp), some significantly declining but still present (~70% coverage) towards the final time-point (including the N and membrane genes), and others with extremely low coverage in the final time-point post diagnosis (e.g. Orf 6,7) (S1 Fig).

## Discussion

This study demonstrates for the first time, that samples in which only the N target is detected by the Allplex assay may contain whole-genome viral sequences that cover overall>70% of the viral genome in all patients. Although viral coverage significantly declined over time, as reflected by the elevated RT-PCR Ct values, the viral genome was still present even in cases considered to be “borderline positive”, i.e. those that are only positive for the N gene target. In fact, genomic coverage differences between the first and final time-points post-diagnosis were insignificant, even with highly stringent criteria for depth of sequencing (X50 depth per position, S3 Table). These results indicate that near-complete/complete viral sequences are present in late time-points post-diagnosis, in at least some patients, and not only residual N-gene segments as initially hypothesized. Nevertheless, the decreased number of reads mapping to the viral genome and high Ct values observed in those late time-points suggest low viral load. Thus, it is plausible that those complete virus genomes present during advanced disease stage do not pose a transmission risk. Indeed, correlations were previously reported between infectivity, the date of disease onset and Ct values [11, 12], suggesting a general cut-off of Ct>30 for non-infectious virus [13, 14]. Furthermore, in rare cases with prolonged shedding of infectious virus, high viral loads (Ct<25) were observed throughout the period of infectivity [15].

In this era, where numerous SARS-CoV-2 diagnostic assays flood laboratories, choosing sensitive and reliable assay is crucial for correct and timely identification and monitoring of infected individuals. The Allplex RT-PCR assay used herein retains high specificity and decreased risk for false negative results by examining three different SARS-CoV-2 targets. Nevertheless, the assay output may be subjected to ambiguous clinical interpretation and subsequent decision-making, when inconclusive results are obtained, such as prolonged exclusive detection of the N gene in recovering patients. The NGS-based findings, which indicate that in the “N-only detected” samples, the E and RdRp genes had near-complete/complete coverage, may suggest reduced primer/probe combination sensitivity for these genes. However, the primers’ locations are not published, hindering efforts to validate the findings in these cases. Therefore, this aspect of the differences in the robustness and sensitivity of the different reactions, was not tested here. The manual of this multiplex assay suggests that the three targets have similar limit of detection (https://www.fda.gov/media/137178). However, limited dilution analysis did show reduction in the capability of detection of the E and RdRp genes and retention of the N signal in few highly diluted positive samples. Nevertheless, further work is underway in our laboratory, to establish the different performance of each reaction within this assay (O Erster, manuscript in preparation).

In summary, this study showed substantial presence of the complete / near complete SARS-CoV-2 genome in nasopharyngeal samples collected up to 35 days post initial diagnosis, where only the N gene was detected by the Allplex assay, highlighting the importance of whole SARS-CoV-2 genome sequencing in directing and monitoring diagnostic assays.

## Supporting information

S1 Table Study samples. Sample details including sample name, date, laboratory of origin, gender, age and Allplex assay resulting Ct values for the N, E and RdRP genes, where “-”denotes an undetected gene. Samples sequenced in this study are marked by *. All samples are patient-matched with at least 2 time-points per patient, where _1, _2, etc. in the sample name denotes the time-points considered for each patient.
(XLSX)

S2 Table End point dilutions of SARS-CoV-2 positive samples. Four SARS-CoV-2 positive samples were diluted with negative sample.
(XLSX)

S3 Table % coverage of SARS-CoV-2 whole genome with varying coverage thresholds. Coverage percentage for the whole genome (WG) and for each gene/segment in all 3 time-points (tp1, tp2, tp3) for each of the 16 patients whose genome was sequenced. Five different coverages are shown, each considering a different threshold for depth of sequencing per position below which “no coverage” was recorded–depths of 1, 5 (used in the study), 10, 20 and 50. For each, the coefficient of determination (r2), the differences in coverage between tp1 and tp3 (Δtp1,3) and the paired T-test p.value of tp1 vs. tp3 (pval(tp1,3) are shown.
(XLSX)

S1 Fig Coverage of SARS-CoV-2 gene across three time-points. Coverage in all three time-points (denoted as 1,2, and 3) are shown for each SARS-CoV-2 gene, with a linear regression line denoting the decline in coverage across the time-points. The coefficient of determination (r2) and the paired t.test p.value of tp1 vs. tp3 are shown.
(TIF)
