# Supplementary figures and images for: Prolonged detection of complete viral genomes demonstrated by SARS-CoV-2 sequencing of serial respiratory specimens
Source: PLoS One. 2021 Aug 5;16(8):e0255691. doi: 10.1371/journal.pone.0255691 (PMC8341697; doi:10.1371/journal.pone.0255691)

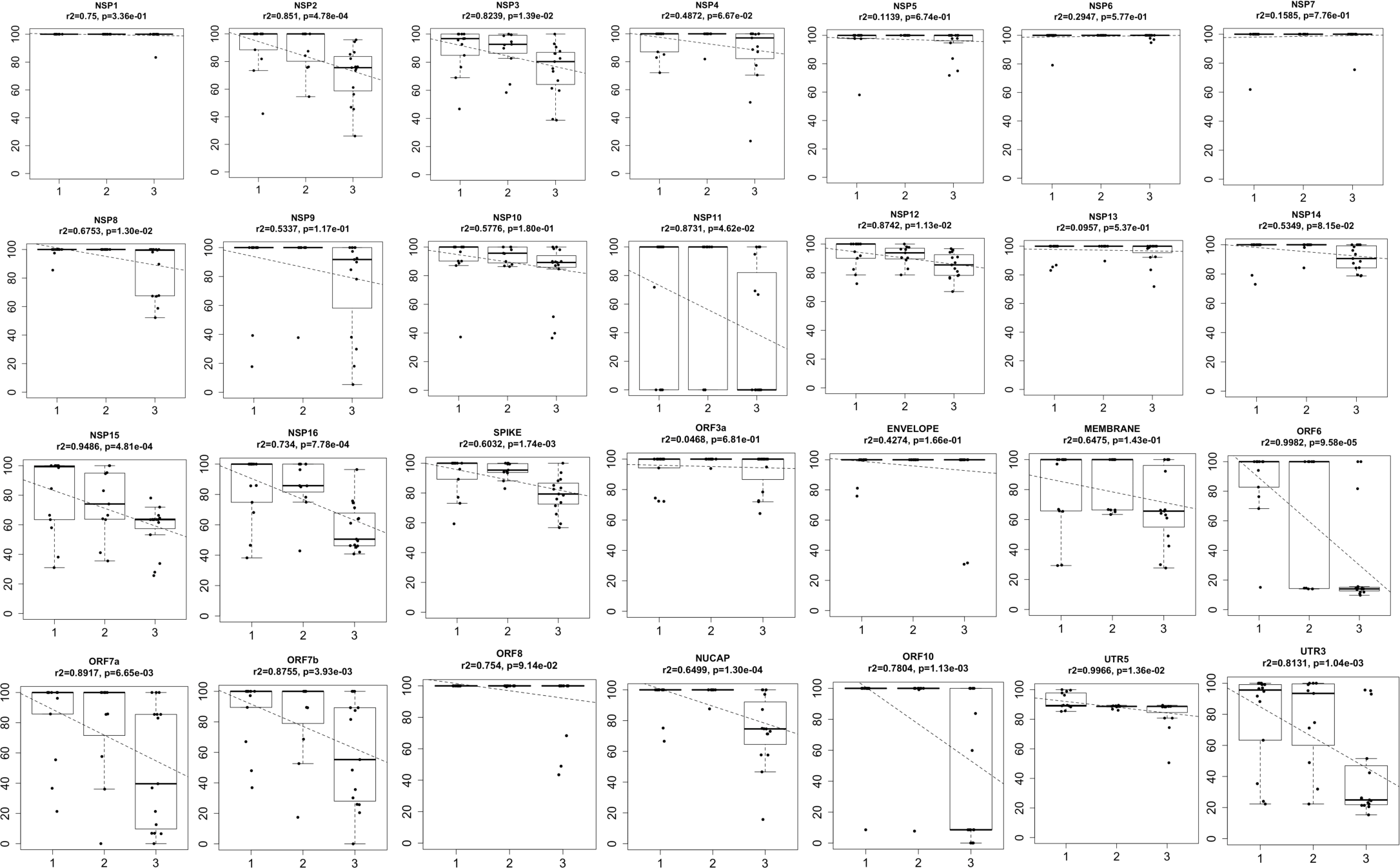

Supplement: S1 Fig — Coverage in all three time-points (denoted as 1,2, and 3) are shown for each SARS-CoV-2 gene, with a linear regression line denoting the decline in coverage across the time-points. The coefficient of determination (r2) and the paired t.test p.value of tp1 vs. tp3 are shown. (TIF) [file pone.0255691.s004.tif]
